# Chit-Chat

**Published:** 1873-07

**Authors:** 


### Editorial Chit Chat.

Dr. Gregg, the chemist, is moving into his new laboratory, on Lake St.

Dixie.—We hear that Dixie is located at Long Beach. We had supposed that the Southern States claimed that honor.

Lady Physician.—The Sultan of Turkey retains the exclusive services of a lady physician—a New Hampshire lady, who graduated in Philadelphia—to attend the females and children of his household.

Cranford, a little village some thirty miles out of New York, on the Central Road of New Jersey, is becoming a popular place for residences of city people. It is said that they get At-water there more conveniently than at most places on that road.

Scranton Times.—We occasionally get a glimpse at this journal and have remarked the vim which has been infused into its local department by its new editor. Ben is a marked success as a local editor, and writes, too, as elegant a magazine article as can be found anywhere.

Personal.—Billy Nicholas, one of the "Lehigh Valley" Conductors, is spending his vacation at Long Beach, capturing sharks. He takes them on a "fly" composed of a five pound hook and ten pounds of pork.

Declines.—A gentleman in this city has been invited to a seat in Prof. Wise's balloon in the attempt which is to be made to cross the Atlantic. Not being a very robust swimmer, and the carrying of a two-masted yacht being inconvenient baggage, he has been compelled to decline the honor.

The River Bank.—It is in a terribly filthy condition. The stench arising therefrom is horrible. Many families occupy the buildings in the locality. Much fatal sickness must occur among them unless means are employed to clean the bank. The Board of Health are endeavoring to abate the nuisance by ordering the removal of all privies emptying into the river. We hope their commands will be enforced.

Chandler, the Artist, is sketching in and about Elmira.

Dr. Johnson said: "A man is, in general, better pleased when he has a good dinner upon his table, than when his wife talks Greek." And we believe him.

Isaac Walton said of strawberries:—"Doubtless God could have made a better berry, but doubtless God never did." It would seem from this that those "old covies" had tastes similar to our own.

New Street Railway.—Dr. Eldridge has determined to build a street railway over the broad avenue, direct to Horseheads. This will afford the people both of this city and of Horseheads, direct communication with the Park, and again illustrates the Doctor's unbounded munificence in contributing his vast wealth to the public interest and enjoyment.

Does Well.—The accomplished Clerk of the Board of Education managed to distribute himself among all our grammar schools at their recent closing exercises, and did not fail to make a neat impromptu speech whenever called upon so to do. He is much interested in school matters and labors dilligently to increase their usefulness.

Troublesome.—Will not our subscribers think far enough, when changing their address, to give us the name of their former post office, as well as that of the new one. If it is desirable to discontinue the Bistoury, they must write us to that effect, or have the Post Master do it for them, and be sure that all arrearages upon the journal are paid. Simply returning a journal marked "refused," will not answer the purpose intended, for we cannot tell from whence it came or who the sender is. Many people are foolish enough to suppose that a publisher can remember all the names of his subscribers as well as their post office address. This might be the case with those publishing a villiage paper, but it cannot be done with a journal of the circulation of the Bistoury. If you want your journal stopped, renewed, or post office changed, write your wishes to the Publisher, being careful to pay up in full what is due the journal, else your desires cannot be complied with.

Our Schools.—It is a real pleasure to attend the closing exercises of our public schools. It is a wonder to us that more people do not avail themselves of the opportunity. Very few parents, we imagine, are familiar with the manner in which our graded schools are conducted, and if they would call occasionally, not only to ascertain how their children are progressing, but to encourage them and the teachers, they would enjoy a very pleasant and instructive visit, and at the same time learn that our school moneys are being invested in a most excellent manner. We have large and substantial school buildings, and intend to have more. With our present corps of teachers, and the system of instruction, we are sure there are no better schools, and very few as good, anywhere in this country.

Our public schools are noted far and wide for their efficiency and most excellent government. The Board of Education are about building two more school houses, which will not only be an ornament but an honor to our city. It is money well invested—no taxes should be more cheerfully paid, for from none do we derive so much benefit.

---

How shall we spend the Sabbath ?—This question is being freely and fully discussed by the clergy of this city upon one side, and the veteran editor of the *Advertiser* on the other. Mr. Fairman has handled the subject in a masterly and logical manner, evincing his capability of making as good a "preacher" as he is an editor. The people as a class, we are of the opinion, endorse Mr. Fairman's view of the subject, believing that the Sabbath is intended for a day of rest and recreation ,and not one to be dedicated wholly to devotional or religious exercises. We are one of those who believe that the Almighty is well served when His creatures praise Him through the enjoyment of His works, and that, being allowed the privilege of breathing the pure air, enjoying the sunshine, the foliage, verdure and other beauties of God's gifts to man, that we are far better prepared to worship and to give Him praise, than when confined within the four walls of a somber meeting-house.—We believe in Eldridge Park meetings, and that where the people love to congregate for health or recreation, there should the minister of God be found, willing and ready to minister to their spiritual wants if called upon so to do.

Passed.—A piscatorial friend of ours was handed the following list of questions in Geography, which were employed in the recent examination of applicants for positions in our common schools. His answers were so good, and his "average" upon examination so high, as to induce the Chairman of the Teacher's Committee to issue him a first class certificate, without need of further examination, to teach any *colored* school in this city. We give below the questions and his answers thereto. The latter will be at once appreciated by all followers of Sir Isaak, in this region:

1. Name four groups of islands washed by the Atlantic Ocean?—Big Island, Clinton, Baldwin, and one at Milan.

2. Which are the principal ranges of mountains in Europe?—Armenia and Ball's Bluff.

3. What possessions has the British Government in Asia?—Not a foot.

4. Which is fartherest west Cape Clear or Cape Finisterre?—Yes.

5. Make a diagram representing the several Zones, and name the lines that separate them?—"Silk, hair and gut," wound about a hat.

6. Name four of the largest cities of the U. S.—Roaring Branch, Ralston, Astonville and Bodines.

7. What large lake or inland sea receives the waters of the largest river in Europe?—Lake Eldridge.

8. What large river flows into the Arctic Ocean?—West Canada Creek.

9. Where are the Philippine Islands?—Just back of Mt. Zoar.

10. What are the most important uses of winds?—To carry your "leader" into tree-tops and so prevent fly-fishing.

---

Artemus Ward.—We have no desire, nor is it our intention to convert this department of the Bistoury into a joker's budget, but the good stories multiply so fast upon us during three months, that we must fire them off or burst. An unpublished story of the genial showman, Artemus Ward, was recently related to us by his busom friend, Mr. E. F. Dixey.—Artemus used to be a constant visitor at Mr. Dixey's dwelling, and frequently sat in the green room of the 11th St. Opera House, Phila., chatting with his friend while he was preparing for the evening performance. One evening, when everything was in readiness to ring up the curtain, Mr. Dixey invited Artemus to step behind the drop-curtain and view the sea of faces that were turned toward the proscenium, the house being packed from pit to dome. Artemus did as he was bidden, and while viewing the house from a small hole in the curtain, that fabric suddenly disappeared aloft, leaving Artemus before an immense audience and in the middle of the stage. This would have been an exceedingly embarrassing position for any one but Artemus Ward, who, after the first surprise at his unexpected appearance upon the stage had subsided, proceeded to deliver himself of a neat little speech, in which he expressed his astonishment at his position and at the proprietors for affording the audience an act not on the bills. After "bringing down the house" handsomely, he introduced Carncross & Dixey's Minstrels, who amused the audience for the balance of the evening.

---

Surprised but not Defeated.—A party of ladies from this city, recently made a raid upon a couple of anglers who had set up house-keeping for themselves, in a tent upon the banks of a beautiful trout-stream. The gentlemen prepared a dinner for the ladies which was pronounced "good"—that is, if the manner in which they dispatched it could be accepted as an indication of their approbation. Either those ladies had purposely starved themselves for three days, else the dinner was amazingly palatable. The only misfortune connected with the affair was that the anglers were compelled to subsist upon short allowance after the exit of the party, until supplies were secured from the city.

---

That Balloon Voyage.—The city papers are discussing the proposed balloon voyage of Prof. Wise across the Atlantic, which he offers to make from Boston, on July 4th. Ten thousand dollars are being collected for the experiment. Prof. King, who made the fine ascension from this city about two years ago, in a letter to one of the Boston journals, argues that the trip cannot be accomplished.—He says a balloon cannot be kept afloat more than 24 hours, and it is of course impossible to make the voyage in that time. Our impression is that if the experiment is tried it would be *wise* to go prepared for a swim in the Atlantic.

